# The degeneration-pain relationship in the temporomandibular joint: Current understandings and rodent models

**DOI:** 10.3389/fpain.2023.1038808

**Published:** 2023-02-09

**Authors:** Man-Kyo Chung, Sheng Wang, Ishraq Alshanqiti, Jiaxin Hu, Jin Y. Ro

**Affiliations:** Department of Neural and Pain Sciences, School of Dentistry, Program in Neuroscience, Center to Advance Chronic Pain Research, University of Maryland Baltimore, Baltimore, MD, United States

**Keywords:** temporomandibular joint, osteoarthritis, degeneration, pain, models

## Abstract

Temporomandibular disorders (TMD) represent a group of musculoskeletal conditions involving the temporomandibular joints (TMJ), the masticatory muscles and associated structures. Painful TMD are highly prevalent and conditions afflict 4% of US adults annually. TMD include heterogenous musculoskeletal pain conditions, such as myalgia, arthralgia, and myofascial pain. A subpopulations of TMD patients show structural changes in TMJ, including disc displacement or degenerative joint diseases (DJD). DJD is a slowly progressing, degenerative disease of the TMJ characterized by cartilage degradation and subchondral bone remodeling. Patients with DJD often develop pain (TMJ osteoarthritis; TMJ OA), but do not always have pain (TMJ osteoarthrosis). Therefore, pain symptoms are not always associated with altered TMJ structures, which suggests that a causal relationship between TMJ degeneration and pain is unclear. Multiple animal models have been developed for determining altered joint structure and pain phenotypes in response to various TMJ injuries. Rodent models of TMJOA and pain include injections to induce inflammation or cartilage destruction, sustained opening of the oral cavity, surgical resection of the articular disc, transgenic approaches to knockout or overexpress key genes, and an integrative approach with superimposed emotional stress or comorbidities. In rodents, TMJ pain and degeneration occur during partially overlapping time periods in these models, which suggests that common biological factors may mediate TMJ pain and degeneration over different time courses. While substances such as intra-articular pro-inflammatory cytokines commonly cause pain and joint degeneration, it remains unclear whether pain or nociceptive activities are causally associated with structural degeneration of TMJ and whether structural degeneration of TMJ is necessary for producing persistent pain. A thorough understanding of the determining factors of pain-structure relationships of TMJ during the onset, progression, and chronification by adopting novel approaches and models should improve the ability to simultaneously treat TMJ pain and TMJ degeneration.

## Introduction

Orofacial pain is a highly prevalent condition. Approximately 15% of the general population experiences various orofacial pain symptoms, such as dento-alveolar pain, tenderness of the temporomandibular joint (TMJ) or masticatory muscles, pain with TMJ biomechanics or pre-auricular pain (1, 2). Temporomandibular disorders (TMD) represent heterogeneous musculoskeletal disorders involving pain conditions, such as myalgia, arthralgia, and myofascial pain, and structural changes, such as disc displacement and joint degeneration (3). A retrospective study involving 4,528 care-seeking TMD patients found that facial pain, ear discomfort, and discomfort or tenderness of the TMJ are typical presenting signs and symptoms of TMD (4). According to the OPPERA (Orofacial Pain: Prospective Evaluation and Risk Assessment) study, 260 people among 2,737 adults aged between 18 and 44 in the United States developed initial TMD during a 2.8 year follow-up period, indicating an incidence of 4% per annum (5). TMD is a multifactorial disease and the mechanisms underlying TMD pain and discomfort are not clearly understood.

TMD also include joint disorders, such as disc displacement and degenerative joint disease (DJD) (3). DJD is defined as a condition whereby articular cartilage deteriorates and the underlying subchondral bone undergoes concomitant remodeling. Radiographic and clinical examinations have shown that approximately 25%–55% of TMD patients have degenerative changes in the TMJ (6–10). DJD includes osteoarthritis and osteoarthrosis. TMJ osteoarthritis (TMJ OA) indicates degenerative changes with pain in the joint whereas TMJ osteoarthrosis is a degenerative condition without any pain-related symptoms. Degenerative changes are more prevalent in female by two folds and increase with aging (9, 11), and could be related to trauma and joint overloading or might be secondary to disc displacement (8, 12–14). However, the majority of TMJOA has a complicated and multiple etiology or is simply idiopathic. Current conservative therapy is successful in managing symptoms, restoring functions, and improving TMJOA (15). However, therapeutic approaches for regenerating the TMJ structures are limited due to a lack of understanding of its pathophysiology and the low healing ability of avascular cartilage (14, 16). It is not clearly known if peripheral pathology, i.e., TMJOA-associated degenerative changes, contributes to TMJ pain. DJD is not likely correlated with the entire spectrum of TMD pain (e.g., myalgia). However, pain from TMJ (arthralgia) is associated with DJD and joint pain is associated with a poor prognosis of TMJOA treatment (15, 17). Therefore, a clear mechanistic understanding of the causal factors associated with joint pain and degeneration should improve our ability to effectively manage the subpopulation of TMD patients with joint pain and TMJOA. In this review, we will focus on the relationship of TMJ degeneration and pain from TMJ rather than other painful TMD conditions, such as myalgia or myofascial pain.

## Degenerative changes and pain in patients with TMJOA

Degenerative changes in the TMJ are highly prevalent in TMD patients (6–10). Radiographic examinations have shown that TMJOA involves the condylar head, articular eminence, and glenoid fossa. Condylar flattening and articular surface erosion are the most commonly observed changes in TMJOA patients (18, 19). While subcortical cysts, surface erosion, osteophytes, or generalized sclerosis are considered as positive criteria for diagnosing TMJOA, flattening of the articular surface and subcortical sclerosis are indeterminate, meaning, an indication of variation of normal (20).

Patients with TMJOA show poorer oral health-related quality of life than healthy individuals (21). Pain from the TMJ is a common and problematic symptom in TMJOA and is more prevalent in females and older adults (22). Self-reported pain from patients with TMJOA is described as dull, heavy, troublesome, and tiring although these are not specific to TMJOA but also reported in other subtypes of TMD patients (22). Pain in the patients with TMJOA often persists and the average pain duration is 6 years (1–408 months) in a study of patients with TMJOA seeking treatment (23). A common symptom of TMJOA is tenderness upon joint palpation. During quantitative sensory testing of the skin overlying the TMJ, TMJOA patients demonstrated distinct sensory phenotypes compared to patients with TMJ pain without degeneration (arthralgia) (24). Specifically, patients with TMJOA had more pronounced mechanical hyperalgesia to blunt pressure compared to those with arthralgia. Furthermore, patients with arthralgia showed reduced sensitivity to both innocuous thermal and mechanical stimuli whereas patients with TMJOA exhibited reduced sensitivity to a tactile stimulus only (24). Another frequent symptom of TMJOA is pain associated with jaw functioning, such as food intake (25). Arthritic TMJs with high inflammatory activity (based on the high concentrations of inflammatory mediators in the synovial fluid) show greater pain intensity on maximum mouth opening and higher pain upon mandibular movements than TMJs with low inflammatory activity (26). However, self-reported jaw pain, function, and disability of patients with DJD are not different from patients with disc displacement (27).

Radiographic studies have supported the association of condyle morphology and destructive condylar changes with pain symptomatology, with condylar flattening being most highly correlated with general pain complaints (19). Condylar erosion has been shown to be proportional to pain intensity, whereas osteophytes seem to be inversely proportional (28). Among patients with arthritic changes of the TMJ, those with pain at rest or during function displayed greater destructive change index and number of CT sections with erosion or subchondral cyst formation than those with no pain (15). In a study involving 89 TMD patients (with TMJOA, TMJ arthralgia, internal derangement, and muscle disorders) and their 178 TMJs, TMJ pain during mandibular movement was correlated with radiographic findings of degenerative changes in the articular surface (erosion, concavity, flattening, osteophytes, osteosclerosis, and subchondral cysts). However, it was not correlated with resorption of the lateral part of the condyle (29). TMJ pain on digital palpation is, however, correlated with both degenerative changes in the articular surfaces and resorption of the lateral part of the condyle. Furthermore, the pressure-pain threshold is lower in patients with resorption of the lateral part of the condyle compared to patients without the condition (29). Three- dimensional surface models from cone-beam CT images show that the condylar morphology of TMJOA with pain is significantly different from asymptomatic subjects: The condyles of TMJOA with pain show resorption of the lateral and medial poles and flattening of the articular surface. Moreover, self-reported pain intensity and duration was correlated with structural changes of the superior surface of the condyles, and pain duration is correlated with structural changes of the superior, posterior, and lateral surfaces of the condyles (23).

However, there are studies suggesting that the extent of degenerative changes in the TMJ is not associated with pain-related variables (6, 9, 30, 31). Several magnetic resonance imaging (MRI) studies focused on the TMJ also have shown equivocal results: In some studies, TMJ pain is associated with severe bone changes, bone marrow edema, and condylar erosion (10, 32–35). In contrast, other MRI studies have indicated that bone does not correlate with pain (30, 36). One clinical study has even suggested that progression of TMJ intra-articular pathologies from disc displacement to degeneration is not correlated with pain, functioning, or disability (37). Similarly, in knee-joint arthritis, knee pain is not strongly correlated with radiographic findings (38, 39). Approximately 15%–76% of patients with knee pain have radiographic knee osteoarthritis (OA), and 15%–81% of patients with radiographic knee OA have pain. However, MRI studies suggest there is a stronger correlation of pain with tissue changes such as synovitis, effusion, and bone marrow lesions (40). Reduction of bone marrow lesions using bisphosphonate is accompanied by reduced pain from knee-joint OA (41). Strontium ranelate is also used to reduce joint degradation and pain from knee-joint OA (42), which suggests a potential contribution of structural changes to pain in this condition.

Lack of unequivocal evidence for the close association of degenerative changes of the TMJ with pain suggests that pain is not a good predictor of degenerative changes or *vice versa*. However, studying the relationship between pain and degenerative changes remains important. Identifying definitive causal factors for joint degeneration and pain can serve as a biomarker of disease onset and progression, which will enable detection and treatment during the early phase of the disease. Persistent pain can adversely impact the prognosis of the treatment of degenerative conditions (15). Novel approaches for pain treatment should not aggravate the degenerative changes either. Therefore, determining factors that commonly or differentially contribute to the onset, progression, and chronification of joint degeneration and pain can help in the development of novel tailored treatments for the different stages of TMJOA and its symptomatology.

## Preclinical studies for TMJOA and pain from TMJ

Multiple animal models have been developed for determining altered joint structures and pain phenotypes in response to TMJ injury. These models are essential for studying the pathogenic mechanisms of TMJOA and exploring effective treatment measures. Compared to human studies, animal models allow for more definitive analyses of cause-and-effect relationships. While there are some morphological dissimilarities between the rodent and human TMJ (e.g., a shallow glenoid fossa and no articular eminence) (43), rodent models of TMJOA and pain should be useful for determining detailed underlying mechanisms. These models typically produce inflammation or biomechanical loading within the TMJ through different methods and evaluate various outcomes, such as degenerative changes of the disc, cartilage or bone of the condyles, and pain-related behaviors. These rodent models have several limitations and there is no ideal model. However, they have provided vast amounts of information concerning neuroimmune, cellular and molecular mechanisms underlying the pathogenesis of the onset, progression, and recovery of TMJ degeneration, as well as peripheral and central plasticity related to pain in inflammatory TMJ. In the future, such studies should lead to the identification of novel biomarkers of disease risk and progression and to explore therapeutic modalities to better manage TMJOA and its relevant symptoms. Animal models can also be used to explore the efficacy or toxicity of new treatments.

TMD patients with pain are heterogeneous in terms of psychosocial and sensory phenotypes and systemic comorbid conditions (44). In addition, the mechanisms underlying the development and maintenance of pain and joint degeneration status are highly diverse. Therefore, to study the relationship between TMJ degeneration and pain, it is likely that no single animal model will ideally mimic the etiology and natural pathogenesis of TMJ DJD translating both condylar degeneration and pain development. Different models may result in a different time course and severity of TMJ degeneration and pain-like behaviors, and their translation into human conditions may be also different in various models. The extent of association between degeneration and pain-like behaviors can also be different. In rodent models of knee OA, models have found different correlations between bone/cartilage damage with pain-like behaviors and nociceptive gene expression in sensory ganglia (45). By critically reviewing the strengths and limitations of each experimental model of TMJOA, an appropriate model can be chosen, or a new model can be developed to test hypotheses involving TMJ-related pain and degeneration.

In addition, mechanistic insights gained through preclinical studies can provide opportunities for developing novel therapeutics to treat both structural changes and pain concurrently or independently. For example, an antibody against disintegrin and metalloproteinase with thrombospondin motifs (ADAMTS-5) decreases both mechanical allodynia and structural changes in a mouse model of knee OA (46). Transforming growth factor–α also decreases cartilage/bone degeneration and knee joint pain (47). Interleukin 6 (IL-6) mediates both cartilage degradation and pain associated with post-traumatic knee OA only in males (48). In contrast, protein kinase Cδ (PKCδ) null mice show reduced joint degeneration in knee OA but display greater pain (47). It is also critical to examine whether molecules enriched in nociceptors regulate joint degeneration since the pain treatment should not adversely impact the pathogenesis of joint degeneration. For example, nerve growth factor-targeted therapy to reduce knee OA pain shows adverse side effects including rapidly progressive OA and osteonecrosis in patients (49). Blockade of voltage-gated Ca^2+^ channels reduces knee arthritis-induced pain, but increases joint destruction (50). Neuropeptides such as substance P and calcitonin gene-related peptide (CGRP) are known to modulate the degeneration of bone and cartilage in a knee OA mouse model (51). Identifying such common and differential mechanisms underlying TMJ degeneration and pain should help in understanding the TMJ degeneration-pain relationship. In the following sections, we will review preclinical studies exploring the mechanisms of TMJ pain and degeneration using various rodent models. We will focus our review on rodent models. Since there are recent outstanding reviews available for rodent models of TMJ degeneration (43, 52, 53), we will focus on the aspects of the TMJ pain-degeneration relationship.

### Injections of inflammatory agents

The etiology of TMJOA is complex and multifactorial, and may be secondary to disc displacement, trauma, parafunctioning, unstable occlusion, or functional overloading (14). These factors can lead to an altered matrix of cartilage, increased joint friction, altered levels of cytokines, and the maintenance of low levels of inflammation to produce focal degeneration. In TMJ synovial fluid, the levels of various proinflammatory and anti-inflammaotry cytokines are differentially altered in TMD patients with or without osseous changes compared to healthy controls (54). In some conditions, such as rheumatoid arthritis, infectious arthritis, or gout, inflammatory activity within the TMJ is high and degenerative changes are more diffuse. Therefore, the direct injection of chemical agents into the TMJ to produce inflammation is the most straightforward method for evoking TMJ degeneration and pain in rodents. Intra-TMJ injections of complete Freund's adjuvant (CFA), zymosan, carrageenan, mustard oil, monoiodo acetate (MIA), albumin, and formalin have been used for this purpose. This approach has been useful for reliably producing measurable nocifensive behaviors, which start quickly (within an hour) and last for a relatively long period (approximately 1–2 weeks). Therefore, intra-TMJ injections have been widely used for studying the mechanisms of TMJ inflammation and the neurobiology of associated acute and chronic pain.

Methods of measuring pain-related outcomes from TMJ have been developed in rodents to mimic various pain conditions frequently reported by TMD patients (Table 1). These assays have also been used to measure pain-related behaviors from craniofacial muscles. Tenderness of the TMJ in patients are evaluated by measuring mechanical sensitivity on the skin overlying the joint using Von Frey filaments, for which a decreased threshold is interpreted as mechanical hyperalgesia (55–57). For mimicking spontaneous pain in TMD patients, head flinching and orofacial rubbing behaviors are assessed in rodents (62, 63, 68, 69). A high facial grimace scale score is also associated with non-evoked, pain-like behaviors after TMJ loading or masseter inflammation (65, 69–71). Preference for a conditioned analgesic indirectly indicates the presence of ongoing pain after inflammation of TMJ or masseter muscle (67, 70). Bite-evoked pain has also been modeled in rats and mice by assessing changes in bite force or gnawing function, for which TMJ or masseter muscle inflammation decreases both (58–60, 69–72). A detailed analysis of meal duration in rats and mice has also shown functional discomfort in the presence of TMJ inflammation (61). Increased central sensitization following prolonged TMJ inflammation can be evaluated by measuring mechanical allodynia from remote sites such as the fore or hindpaw (67). Increased anxiety in TMD patients has also been modeled in rodents using anxiety-like behavioral assays such as the elevated plus maze and open field tests (66). The duration and extent of nocifensive behaviors are varied and depend on multiple factors including the agents used for producing TMJ inflammation, the behavioral assays used, and the strain, species, and sex of the animals.

**Table 1:** Temporomandibular joint pain assays in rodents.

Assay	Species	Outcomes related to hyperalgesia	Relevant clinical pain conditions	References
Von Frey skin test on the TMJ or masseter muscle	R, M	Decreased threshold	Tenderness	(55–57)
Bite force	M	Decreased force	Bite-evoked pain	(58, 59)
Dolognawmeter	M	Increased break time	Bite-evoked pain	(60)
Meal pattern	R, M	Increased meal duration	Bite-evoked pain	(61)
Facial rubbing and grooming	R, M	Increased flinching and rubbing	Spontaneous pain	(62–64)
Grimace scale	R, M	Increased score	Spontaneous pain	(56, 65)
Anxiety-like behaviors	R	Open field test Elevated plus maze	Anxiety and affective pain	(66)
Conditioned place preference	R	Increased preference to a conditioned analgesic	Spontaneous pain	(67)
Von Frey test on remote site	R	Decreased threshold	Widespread pain	(67)

R, rats; M, mice.

**Figure 1:**
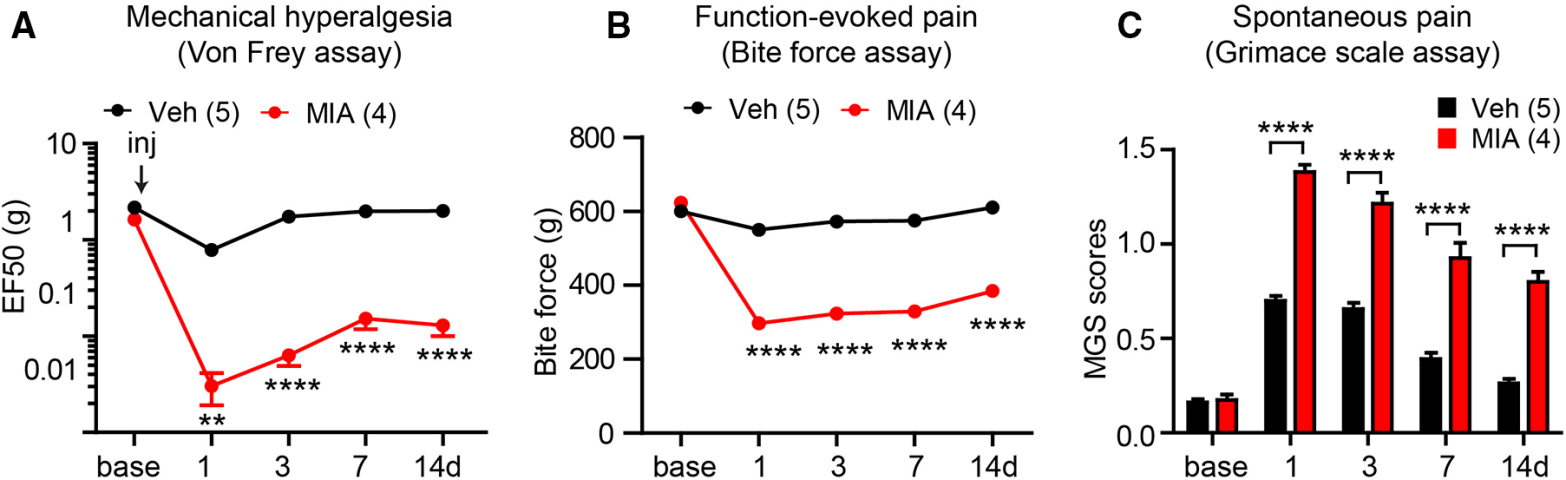
Examples of measuring different types of persistent pain induced by temporomandibular joint (TMJ) inflammation in mice. Bilateral TMJs of C57bl/6 mice were injected with monoiodo acetate (MIA). Controls were injected with saline. Mechanical sensitivity of the skin over the right TMJ using the Von Frey assay (**A**), bite-force (**B**), and mouse grimace scale (MGS) (**C**) were determined. **p < 0.01; ****p < 0.0001 in Bonferroni post-tests following two-way repeated measure ANOVAs. We followed methods described previously (69, 70, 75).

Intra-TMJ injections of CFA induces mechanical hyperalgesia on the TMJ, which peaks after 1–4 days and lasts approximately 10–14 days in mice and rats (56, 57). Intra-TMJ CFA administration increase meal durations for 19 days in rats and 1 week in DBA/1LacJ mice, but does not affect C57bl/6 mice (61). Intra-TMJ CFA also reduces bite force for 1–9 days (59, 73) and increases the mouse grimace scale score for 2–3 days (56). Following MIA injection, mechanical sensitivity on TMJ skin increases from day 1 and lasts for 2 weeks (67, 74). This mechanical hyperalgesia declines from baseline at 3 weeks with recovery occurring after 4 weeks (74). MIA-injected rats display a conditioned place preference for the systemic administration of duloxetine at 7 days. They also exhibit increased feeding duration and total number of meals, suggesting the presence of ongoing pain (67). It appears that MIA tends to produce a longer duration of hyperalgesia than CFA. In addition, when it comes to injection method, most studies focused on degeneration and mechanism, while the studies of phenotype of pain are limited. Therefore, we conducted an example of phenotype experiment to figure out its effectiveness. Intra-TMJ injections of MIA increased mechanical sensitivity and mouse grimace scale scores and decreased the bite force for at least 2 weeks in mice (Figure 1). Establishing the mouse model of persistent TMJ pain can facilitate mechanistic study using a variety of transgenic models.

Studies using models that involve the artificial inflammation of the TMJ and other orofacial areas have led to important discoveries concerning the neurophysiological mechanisms in primary afferents and central neural transmissions associated with pain from the TMJ and other oral and craniofacial regions. These studies have laid a solid foundation for our current understanding of orofacial pain mechanisms. While a detailed discussion of these mechanisms is beyond the scope of this review, there are other publications that have reviewed these topics (57, 76–82). In brief, the identity of the subtypes of joint nociceptors and the peripheral sensitization of joint afferents leading to hypersensitivity has been defined (83), as has the dominant role TRPV1-expressing afferents play in TMJ pain (84). Furthermore, the causative role of pronociceptive ion channels in nociceptors (e.g., TRPV4 or Nav1.7) has been identified (58, 85). The anti-nociceptive role of opioids, cannabinoids, and β adrenergic receptors are also known (63, 86–88). Highly convergent nociceptive inputs from the TMJ and other orofacial tissues (such as skin, tooth pulp, and the masseter muscle) on dorsal horn neurons in the cervical spinal cord and trigeminal subnucleus caudalis (Vc) may underlie the diffuse and widespread nature of pain from TMD (89–92). Central sensitization mechanisms involving NMDA receptors, MARK, and non-neuronal cells (e.g., microglia and astrocytes) in the medullary dorsal horn are well known (93–96). Intraganglionic mechanisms can also mediate widespread pain *via* the spreading of nociceptive signals within the trigeminal ganglia (TG) through soluble mediators and gap junction channels involving satellite glia (97–99). Determining the mechanisms underlying sex differences has also been at the center of investigational interest. Sex hormones (e.g., estrogen, progesterone, and testosterone) differentially affect inflammatory responses in the TMJ (100–103) and the activities of joint afferents (104). Currently, sex differences and the influence of gonadal hormones on nocifensive behaviors have been determined (105–107), and the roles of sex hormones on the regulation of neuron activity in the Vc and upper cervical spinal cord are also well established (108, 109).

Injecting chemical agents into the TMJ has also been widely used to determine the mechanisms of inflammation-induced TMJ degeneration. CFA contains heat-killed mycobacteria that produces immune responses and inflammation, which recapitulates microbial infection-like acute inflammatory responses. Two weeks after intra-TMJ injections of CFA in mice, the TMJ showed deposition of extracellular matrix, such as aggrecan and collagen type II, and soft tissue changes that include pannus formation in TMJ disc attachments and synovitis with infiltration of inflammatory cells (110). However, cartilage degradation or bony changes do not occur, which suggests that this model cannot represent pathogenesis during the chronic phase of OA. Another study in rats demonstrated that intra-TMJ CFA injections induced histological changes in cartilage and subchondral bone that peaked after 2 weeks, but recovered thereafter without progressive, long-term changes (111). When CFA injections were administered into rat TMJs two weeks apart, inflammation in the synovium was sustained up to 5 weeks after the 1st CFA injection, and the TMJ disc was thickened with increased cellularity (112). However, there were no changes in the condylar cartilage or the subchondral bone. In these experiments, CFA was injected into the upper joint cavity. In contrast, injection of CFA into the lower joint cavity induced radiographic and histologic bony changes within 14 days (113), supporting the idea that CFA does not induce degenerative changes of the disc.

MIA has also been widely used to model degeneration and pain from knee joint OA in rodents (67, 74, 114, 115). MIA is an inhibitor of glyceraldehyde-3-phosphate dehydrogenase and interferes with the metabolism of glycolysis in nerves and chondrocytes (116, 117). Injections of 0.5 mg MIA into the upper compartment of the TMJ in female rats induces ultrastructural changes in chondrocytes and the disc as early as day 1. Chondrocytes are nearly lost in the cartilage of the temporal fossa and intermediate zone of the disc after 3 days. Exudates in the upper compartment is evident within 1 week, and infiltrated immune cells are present within 2 weeks. In µCT, changes in the condylar surface appear within 1 week, while erosion and defects are evident within 2–4 weeks. Sclerotic changes begin by 8 weeks (74). Hence, MIA may produce more global and long-lasting degenerative changes in the TMJ compared to CFA.

Although the injection of exogenous substances into the TMJ consequently produces both pain and degenerations, the model cannot ideally mimic the unknown real factors contributing to the progression and the maintenance of persistent pain and degeneration in humans. Despite the limitation, direct injection chemical agents can be still useful for understanding inflammatory processes leading to TMJ pain and degeneration.

### Forced mouth opening

The TMJ is unique in its high mobility compared to other joints. Mandibular condyles not only make a hinge movement, but also rotate horizontally during mastication and glide over the temporal bone upon wide opening of the mouth. The structure of the TMJ has evolved to accommodate the wide and dynamic range of movements with high functionality and maximum efficiency. However, excessive functioning of the TMJ beyond its physiological limits can be traumatic and leads to the development of chronic pain and structural remodeling of the joint. Indeed, there is a strong association between prolonged mouth opening and the development of chronic TMD. In one prospective study, the TMD annual incidence was doubled in patients who had experienced a jaw injury (118). Both extrinsic injuries (e.g., tooth extractions or dental treatments, oral intubation, sports injury, or motor vehicle accidents) or intrinsic injuries (e.g., yawning or sustained mouth opening) can be risk factors for TMD. Interestingly, the association of jaw injury with painful TMD are higher in patients with increased heat pain (119). Not only pain, jaw trauma can also lead to the degenerative changes of TMJ (13, 120). Therefore, it is important to investigate pain responses and TMJ structure following TMJ trauma or injury. To achieve this, animal models have been developed for delivering intrinsic injuries to the TMJ by adding abnormal TMJ loading or inducing a non-physiological range of jaw openings (121–123). These models are non-invasive and do not involve open trauma or injecting artificial chemicals into the joint to cause structural destruction.

A single event of prolonged jaw opening for 20 min in rats induces increased head withdrawal to mechanical stimuli as early as 2 h after the event, which then lasts up to 7 days (123). The procedure increases pro-inflammatory cytokines in the TG and upper cervical spinal cord up to 14 days after induction (123). Sustained and repeated mouth opening (60 min/day for 7 days) in rats by applying 2 N to the jaw has produced mechanical hyperalgesia on the face as early as day 1 and has lasted for 3 days after termination of the induction. When loading force was increased to 3.5 N, mechanical hyperalgesia was not resolved—even after a week following the termination of the induction (122, 124). The rat grimace scale score was increased in the 3.5 N group, but not in the 2 N group, over a period of 1–7 days following the initiation of sustained mouth opening, during which condylar cartilage was not affected (65). However, damage to the condylar cartilage was evident on day 15. Both the 2 N and 3.5 N groups showed thinning of the condylar cartilage with irregular chondrocyte organization and there was no significant difference between two groups. However, the expression of MMP-13, hypoxia-inducible factor 1α (HIF-1α, and TNF in the condylar cartilage were higher in the group with greater loading (122). A preemptive, single intra-TMJ injection of etanercept (a biologic TNF inhibitor) decreased mechanical hyperalgesia and rat grimace scale scores until day 7 following the initiation of TMJ loading (125). Concomitantly, an intra-TMJ injection of etanercept reduced HIF-2α expression and hypoxia in TMJ cartilage on day 8 (125). Detailed investigations of the pericellular matrix surrounding chondrocytes within the TMJ cartilage following loading showed that collagen VI increased in both the 2 N and 3.5 N groups, whereas there was a greater increase in aggrecan non-epitopes in the 3.5 N group. These results suggest that aggrecan fragmentation may be relevant to pain responses and the severity of structural damage to the TMJ (126).

In C57BL/6 mice, sustained passive mouth opening by 11.5 mm (1.5 h per day for 5 consecutive days) produced mechanical hyperalgesia of the masseter muscle, which began as early as 1 day after the 1st opening event and lasted for 32 days afterwards (127). The amount of hard pellet food intake was also reduced. Sustained mouth opening increased the infiltration of macrophages into the TG and the activation of microglia in the Vc. Inhibition of colony stimulating factor 1 (CSF1) receptor reduced this infiltration into the TG, TMJ, and masseter muscles and decreased the activation of microglia in the Vc. Importantly, the CSF1 inhibitor significantly attenuated the development of mechanical hyperalgesia by sustained mouth opening, suggesting macrophages contribute to the peripheral sensitization of trigeminal nociceptors from TMJ injury. Sustained mouth opening also led to subtle changes in the condylar cartilage on day 5.

Mechanisms of TMJ structural degeneration after forced mouth opening in mice were studied in a sustained mouth-opening model that used a spring to maintain an open mouth by 14 mm and delivered 2 N force for 3 h per day for 5 days (128). Four weeks after the completion of the force loading sessions, destruction of TMJ cartilage and subchondral bone was obvious on mCT and in histological assays (128). Rebamipide, an anti-oxidant and anti-inflammatory agent, reduced the TMJ degeneration with reduced osteoclastogenesis (128). Although the effects of rebamipide on pain were not assessed in that study, the intra-articular injection of rebamipide into the knee joint attenuates hyperalgesia and cartilage degeneration following MIA injection (129).

Rodent forced mouth opening models can be useful for determining structural changes in the TMJ that mimic loading and trauma pathogenesis in humans. The model has translational potential since the associations of TMJ trauma with the development of painful TMD or TMJ degeneration is known (13, 118). Determining pain phenotypes using various assays during the time course of the pathogenesis of TMJOA and a more careful assessment of pain and TMJ degeneration over longer periods is necessary.

Of note, the non-physiological range of excessive occlusal interferences can lead to pain and TMJ degeneration in animals as reviewed previously (52, 130). However, it is important to emphasize that the animal models involving occlusal interference are built on a highly controversial assumption that occlusion is an etiologic factor leading to TMD development, but the evidence supporting this assumption is not established (131). There is a growing consensus among TMD experts that occlusion should not be considered a contributing factor for the common TMD (132). Therefore, translational potential of the models involving occlusion alteration is weak and we do not discuss this model in this review.

### Surgical approaches

A surgical approach to destabilize the knee joint by transection of the medial meniscotibial ligament has been widely used in rodents to produce slow progression of structural changes and pain-like behaviors from 4 weeks following surgery (133, 134). Degeneration of the knee joint is accompanied by the development of pain-like behaviors such as weight bearing asymmetry or decreased paw withdrawal thresholds. Despite their correlation, it is still unclear if the extent of degeneration of the knee joint and pain-like behaviors are causative or mechanistically associated. For example, a modified surgical technique only attenuates pain-like behaviors after 13 weeks following surgery but did not affect joint pathology (135). However, comparable artificial surgical impairments of TMJ stability have been used to produce TMJOA in rodents (Table 2). Removal of the entire or a portion of the articular disc can affect the ability of the condylar surface of the TMJ to withstand forces and absorb loads, which could result in excessive mechanical forces on the condylar surface over a small area leading to TMJOA.

**Table 2:** Examples of surgical procedures to induce temporomandibular joint osteoarthritis in rodents.

Type of surgery	Species	Euthanized time point (post-operatively)	Reference
Discectomy	R, M	9, 16, 28 days (rats) 1, 2, 4, 6 weeks (mice)	(136–138)
Partial discectomy	M	2, 4, 8, 12, 16 weeks	(139, 140)
Disc perforation	R	1, 4 weeks	(141)
Disc displacement	R	1, 4, 8 weeks/15, 30, 60 days	(142, 143)

R, rats; M, mice.

Partial or total removal of the disc in the TMJ of rats and mice results in early-onset OA-like changes (136, 137, 139, 144). Partial discectomy that removes the lateral part of the disc of the TMJ in C67BL/6 mice produces alterations in the condylar cartilage as early as 4 weeks following surgery. At 16 weeks, destruction of the cartilage was more evident, but subchondral bone was not affected. Surgical perforation of the posterolateral portion of the TMJ disc in rats has led to the destruction of cartilage condyles and subchondral bone at 4 weeks (145). Intra-TMJ administration of interleukin 37b, an isoform of the anti-inflammatory cytokine interleukin 37, decreased post-surgical degenerative changes of the TMJ (145). Although pain phenotypes were not determined in the rat model, patients with TMJ synovitis exhibit a high expression of interleukin 37 in synovial fluid, which is correlated with self-reported pain levels (145). Surgical anterior displacement of the TMJ disc in rats also produces OA-like alterations in TMJ structure (142, 143), which is apparently less severe than other surgical approaches. Although pain-like behaviors were not directly measured, no visible behavioral changes or stress-related signs were reported.

Surgical models of TMJOA are primarily used for studying TMJ degeneration rather than pain phenotypes, and information regarding the correlation or association of pain and TMJ structure is unavailable. Since surgical manipulation itself can cause inflammation inside the affected joint, segregating the effects of acute, post-operational changes on TMJ structure from the effects of surgical manipulation of the disc is difficult. Interestingly, unilateral, partial discectomy not only leads to degeneration in the surgical-side of the TMJ, but also initiates early-onset articular cartilage degeneration in the contralateral, non-surgical side of the TMJ in mice (140). Therefore, the contralateral non-surgical side of the TMJ might serve as a model of TMJ degeneration that mimics the natural progression of TMJOA in patients. Nonetheless, differentiation of post-operative pain from the pain caused by structural alterations relevant to TMJOA can be challenging, which might be a limitation when using surgical models for determining the pain-degeneration relationship.

### Genetic models of TMJOA

Mice carrying spontaneous or targeted mutations of multiple genes naturally develop TMJ degeneration in a non-invasive manner and without the need for injecting agents or inducing trauma (Table 3). These mouse models have been used to determine molecular contributions to the development and degeneration of the TMJ, which has been recently reviewed elsewhere (43). The mouse genetic model has deepened our understanding of the causal contributions of matrix proteins, transcription factors, and regulatory signaling such as growth factors. Mouse models that carry natural mutations of genes encoding matrix proteins have been useful for understanding their critical contributions to skeletal anomalies associated with human disease. For example, the disproportionate micromelia (Dmm) mutation is associated with mutations of Col2a1 that encodes collagen type II, which results in condylar cartilage abnormalities in the TMJ (147). Dmm TMJs exhibit degeneration of the condylar cartilage from 6 months of age, which continues to progress until 12 months of age. During the progression, expression markers, such as HtrA1, Ddr2 and Mmp13 were detected (147). The chondrodysplasia (Cho) mutation is caused by a single-nucleotide deletion of Col11a1, which encodes for collagen type XI (146). In the TMJ of Cho mice, changes in proteoglycans occur as early as 3 months and fissures of the condylar cartilage were evident at 9 month of age. Targeted mutations of multiple genes has also uncovered their roles in TMJOA. Transgenic mice expressing the deletion mutant of Col2a1 (Del1 mice) show defects in condylar cartilage as early as 3 months and progression of degenerative changes occurs until 15 months of age (148). Mice with double mutations of 2 genes encoding proteoglycans, Bgn (biglycan) and Fmod (fromodulin) develop degenerative changes at 6 months and show complete destruction of the TMJ at 18 months (149). Knockouts of proteoglycan 4 (Prg4), which encodes lubricin, which functions as a lubricant inside the TMJ, induces early signs of OA-like degenerative changes including increased thickness of the glenoid fossa, articular disc, and condylar head as early as 3 months. At 12 months, morphological and osseous abnormalities of the condyle was evident in radiographic analyses (150). Knockouts of discoidin domain receptor 1, a tyrosine kinase receptor, develop osteoarthritic degenerative changes of the TMJ as early as 9 weeks of age (151).

**Table 3:** Genetic mouse models producing temporomandibular joint (TMJ) osteoarthritis-like phenotypes.

Model	Gene mutation or manipulation	Time course for TMJ degeneration	References
chondrodysplasia (Cho) mutation	A single-nucleotide deletion of Col11a1	After 3 months of age	(146)
disproportionate micromelia (Dmm) mutation	Mutations of Col2a1	After 6 months of age	(147)
Del1 mice	Deletion mutation of Col2a1	After 3 months of age	(148)
Bgn^−/0^Fmod^−/−^ mice	Double mutation of two genes encoding proteoglycans, Bgn (biglycan) and Fmod (fromodulin)	After 6 months of age	(149)
Prg4–/–mice	Knockout of proteoglycan 4, which encodes lubricin	After 3 months of age	(150)
DDR1–/–mice	Knockout of discoidin domain receptor 1, a tyrosine kinase receptor	After 9 weeks of age	(151)
IL1β overexpression in the TMJ	Cre-dependent conditional expression of human IL1β	At 8 weeks following induction	(64)
TNF overexpression	TNF overexpression	At 12 weeks of age	(152)

Genetic models involving pro-inflammatory cytokines have contributed to thorough validations of their role in TMJOA pain and degeneration. Pro-inflammatory cytokines such as IL1β, TNF, and IL6 are elevated in the TMJs of patients with TMD (153). The IL1β level in TMJ synovial fluid from TMD patients is associated with pain and tenderness on palpation (154). Moreover, pain on TMJ palpation and mandibular movement is associated with increased levels of TNF in TMJ synovial fluid (155). Furthermore, intra-TMJ CFA injections elevate TNF and IL1β inside the joint (156). Sufficiency of IL1β in TMJ degeneration and pain was convincingly shown using a model that overexpressed the IL1β gene under the Col1a1 promoter within the TMJ (by intra-TMJ injection of the viral vector encoding Cre). In this model, joint degenerative changes were evident 8 weeks following Cre injection (157). Increases of CGRP in the TG and nocifensive behaviors such as facial grooming also occurred (157). Nocifensive behaviors in this model were attenuated by virally mediated overexpression of the mu-opioid receptor gene in the TMJ (64). IL1β plays an important role in the medullary dorsal horn (158), and TMJ degeneration *via* overexpression of IL1β is prevented by the overexpression of the IL1 receptor antagonist in the brainstem. Consistently, conditional overexpression of IL1β in the medullary dorsal horn driven GFAP promoter is sufficient to induce TMJ degeneration, albeit to a lesser extent than the overexpression of IL1β in the TMJ. Furthermore, facial grooming behaviors induced by the overexpression of IL1β in the TMJ is attenuated by the overexpression of the IL1 receptor antagonist in the brainstem (158). These results suggest that IL1β in both the TMJ and the medullary dorsal horn contribute to TMJ degeneration and pain.

TNF also contributes to TMJ pain. TNF is released not only by immune cells but also by the glia and neurons of the peripheral and central nervous systems (159). Overexpression of TNF is sufficient to develop OA changes in the TMJ such as articular and condylar bony changes, an influx of inflammatory cells in the synovium, and fissuring of the condylar surface at 12 weeks of age (152). TNF overexpression also causes severe destruction of the condyle, with condylar surface erosion, bone resorption, and loss of the disc at 14 weeks (160). Although pain phenotypes were not evaluated in this model, the contribution of TNFα to pain from rodent models of TMJ inflammation is known. For example, intra-TMJ TNF injections increases head withdrawal responses (161). Intra-TMJ CFA increases TNF in the TMJ, the TG, and the Vc (56, 162). Furthermore, CFA-induced nocifensive behaviors (mechanical hyperalgesia of facial skin and increased mouse grimace scale scores) are attenuated in mice lacking TNF (56).

These genetic mouse models are useful for determining the molecular contributors to degeneration of the TMJ that mimic natural pathogenesis in humans (with an onset at 2 to 6 months of age and further progression with aging). However, most studies are only focused on the time course of the disease and biological factors involved in degenerative changes within the TMJ and, therefore, do not pay much attention to pain-related phenotypes. Hence, determining pain phenotypes using the various assays discussed above during the time course of pathogenesis of TMJOA in these genetic models is warranted.

### Models involving emotional stress and comorbid conditions

International Association for the Study of Pain defines pain as “an unpleasant sensory and emotional experience associated with, or resembling that associated with, actual or potential tissue damage, or described in terms of such damage”, meaning pain is not a simple sensation but involves emotional aspects. One prospective study showed that multiple psychosocial variables including somatic symptoms, general psychological symptoms, a negative mood, and multiple measures of stress are risk factors for the onset of painful TMD (163). Furthermore, in an unbiased clustering of subjects based on biopsychosocial measurements, a cluster showing greater pain sensitivity and greater psychological distress had a greater risk of painful TMD onset, as well as other comorbid pain conditions such as chronic headache, low-back pain, and irritable bowel syndrome (44). Therefore, chronic pain from TMD should be under the influence of psychosocial factors, although relationship between the psychosocial factors and pain associated with TMJOA is not clear. As surrogate models in rodents, the effects of psychological stress on TMJ pain have been actively studied. Intra-TMJ CFA injections increases anxiety-like behaviors (reduced entry into open arms in elevated plus mazes and light boxes in light/dark box assays) (66). Rats exposed to chronic restraint stress exhibit greater anxiety-like behavior and formalin-induced nocifensive behaviors (164). Stress-induced hyperalgesia from the TMJ is apparently derived from both peripheral and central mechanisms, while sleep deprivation increases inflammatory cytokines from the synovial membrane (165). Stress also enhances the activity of TMJ neurons in the deep lamina of the Vc and enhances the integration of nociceptive information from the masseter muscles (166). Interestingly, psychological stress also affects condyle structure (167). When rats experience psychological stress by visual, olfactory, and auditory stimuli from other rats receiving electric foot shocks in an adjacent chamber, anxiety-like behaviors increase. Furthermore, condylar cartilage and subchondral bony changes occur as early as 3 weeks, which is accompanied by increased expression of MMP3, IL1β and TNF in the TMJ (168, 169). Adrenergic signaling through α2 receptors in chondrocytes promotes TMJ degeneration (170). Chronic sleep deprivation activates extracellular, signal-regulated kinases (ERK) in the TMJ, and inhibition of ERK reduces the expression of matrix metalloproteinases genes and decreases pathological changes of condyle (171). Therefore, chronic stress likely affects both TMJ degeneration and pain.

The mechanisms of TMD and comorbid pain conditions, such as irritable bowel syndrome, have also been studied using rodent models. Masseter inflammation followed by swim stress induces months-long visceral hypersensitivity and concomitant cutaneous hyperalgesia in rats, which is mediated by ERK1/2 in the spinal cord (172, 173). Such sensitization between orofacial and gut tissues were also studied in another mouse model called the “double-hit model” (174). Intra-TMJ CFA injections produced mechanical hypersensitivity of the skin overlying the TMJ, which recovered within a week. After 3 weeks, colonic injections of mustard oil produced no hypersensitivity in wild type mice. In contrast, TNFR1/R2 double knockout mice developed mechanical hypersensitivity on the skin overlying the TMJ for up to 18 weeks. Systemic antagonism of TNF reverses mechanical hyperalgesia that has developed after colonic mustard oil (174), suggesting the role of circulating cytokines in trans-segmental sensitization. A recent study has reported a more sophisticated comorbid pain model: unilateral anterior cross bite in rats produces somatic pain hypersensitivity, which involves the cholecystokinin receptor-mediated descending facilitation system (175). Mechanistically, the gut microbiome was also proposed to be involved in TMJ pain (176). Intra-TMJ CFA injections induced mechanical hyperalgesia in the facial skin and alterations of the gut microbiota. This was reversed by systemic treatment with resveratrol and *via* transplantation of fecal microbiota from resveratrol-treated mice into mice with intra-TMJ CFA (176), which suggests a contribution of gut microbial dysbiosis to TMJ pain. In humans, an abundance of gut *Streptococcus* is associated with pain and inflammation in knee OA (177). While a microbiota-skeletal axis has been proposed, determining the association of TMJOA and pain with the gut microbiome and comorbid pain conditions is warranted.

## Conclusion

Studies that have focused on the mechanisms of TMJ pain and structural degeneration using different models indicate that TMJ pain and degeneration occur over a partially overlapping time course. There are molecules that commonly cause pain and joint degeneration, e.g., intra-articular pro-inflammatory cytokines. However, it is unclear whether pain is causally associated with structural degeneration and whether structural degeneration is necessary for producing persistent pain. To experimentally address the pain-structure relationship, it will be important to determine whether the activity of peripheral and central pathways involved in TMJ pain contributes to TMJ degeneration or *vice versa*. Considering the broad impact of the brain on skeletal homeostasis (178), it is likely that nociceptors and brain plasticity associated with pain upon TMJ injury can modulate the degeneration of the TMJ. More detailed investigations of the pain-structure relationship in the TMJ can suggest new therapeutic options that treat pain without adverse effects on the TMJ structure. To determine the mechanistic association of pain and TMJ structure, it is important to select the right animal models. However, TMD are multifactorial conditions, and it may be that a single animal model cannot represent all aspects of pain and the structural changes in the TMJ with DJD. As different animal models mimic some aspects of the signs and symptoms of TMJOA patients, careful selection of one or combinations of animal models will be critical to meet the goal of investigations. Further development of non-invasive models mimicking natural progress of degenerative changes in TMJ with persistent pain conditions and comorbidities can improve the mechanistic understanding and the development of novel strategies for better managements of TMD.
